# Extending the GMR Current Measurement Range with a Counteracting Magnetic Field

**DOI:** 10.3390/s130608042

**Published:** 2013-06-21

**Authors:** Tin Yan Poon, Norman Chung Fai Tse, Ricky Wing Hong Lau

**Affiliations:** 1 Division of Building Science and Technology, City University of Hong Kong, Hong Kong; E-Mail: bsnorman@cityu.edu.hk; 2 Department of Electronic Engineering, City University of Hong Kong, Hong Kong; E-Mail: itwhlau@cityu.edu.hk

**Keywords:** giant magnetoresistance, current sensor, linearization, physical sensor, magnetic field sensor, Helmholtz coil

## Abstract

Traditionally, current transformers are often used for current measurement in low voltage (LV) electrical networks. They have a large physical size and are not designed for use with power electronic circuits. Semiconductor-based current sensing devices such as the Hall sensor and Giant Magnetoresistive (GMR) sensor are advantageous in terms of small size, high sensitivity, wide frequency range, low power consumption, and relatively low cost. Nevertheless, the operational characteristics of these devices limit their current measurement range. In this paper, a design based on using counteracting magnetic field is introduced for extending the GMR current measurement range from 9 A (unipolar) to ±45 A. A prototype has been implemented to verify the design and the linear operation of the circuit is demonstrated by experimental results. A microcontroller unit (MCU) is used to provide an automatic scaling function to optimize the performance of the proposed current sensor.

## 1. Introduction

Current measurement is essential in modern electrical system applications and different current sensing devices have been developed to cater to various needs. Magnetic sensors have been widely used in current measurements since they are non-intrusive and provide galvanic isolation. Typical current sensors include the current transformer, Hall sensor, optical current sensor, anisotropic magnetoresistive (AMR) sensor and GMR sensor [1]. Various methods have also been developed to improve the linearity, and frequency response of these devices [2].

Many GMR-based sensors have been developed by researchers ever since the giant magnetoresistance effect was discovered in 1988 [3–5]. GMR sensors offer high sensitivity, wide frequency range, small size, low power consumption and they are compatible with many other state-of-the-art technologies [6,7]. GMR sensors have been employed in various applications, such as current sensing in low field, speed sensing and read head of hard drives [8,9]. However, as the linearity range of GMR sensors is narrow compared with other sensors such as Hall sensors, GMR sensors are not suitable for large current sensing [10,11]. In addition, the output of some of GMR sensors is unipolar, which limits its application in AC measurements [12].

Much research has been done to improve the linearity of GMR. In [13], a closed-loop operation is used to improve the linearity of a Hall sensor or magnetoresistive sensor. In [14], low frequency capture is used to extend a GMR sensor to ±800 A. In [10], linearity is improved by hysteresis modeling compensation. In [6], an automatic procedure for calibration and adjustment is used to reduce hysteresis and temperature dependence.

In this paper, the authors propose a technique to extend the linear range of the GMR current sensor and to convert unipolar output into bipolar output so that the GMR sensor can be applied to both DC and AC measurements, mainly for power quality (PQ) measurement in building LV electrical networks. The basic approach is to use the Helmholtz coil to generate a magnetic field to counteract the magnetic flux produced by the current carrying wire/cable. In such a way, the magnetic flux passing through the GMR is reduced and the current range is effectively increased. The polarity conversion is simply achieved by using current biasing. With a sufficiently high gain of the power amplifier, the output current is proportional to the input current. A prototype has been implemented and experimental results demonstrate that the current measurement range can be extended from 9 A (unipolar) to ±45 A. The design is further improved by an automatic scaling circuit controlled by a MCU which minimizes the gain of the power amplifier circuit so as to optimize the frequency response. Compared to previous works, the main advantages of the proposed approach are: (1) with the use of Helmholtz coil, the effect of inaccurate positioning of GMR on the measurement result is minimized even if a magnetic core is not provided; (2) the performance of the sensor is optimized by the automatic gain scaling function.

The paper is arranged as follows: Section 2 introduces some background information of the GMR. Section 3 presents the Helmholtz coil that is used as the feedback coils for magnetic field counteraction. Section 4 discusses the design theory and the transfer function for the sensor design. Section 5 presents the design characteristics. Section 6 presents the detailed construction of the prototype sensor. Section 7 presents the performance results of the prototype sensor. Section 8 discusses the automatic gain control circuit for improving the performance of the GMR sensor. Section 9 presents the detailed construction of the improved sensor circuit design. Section 10 presents the performance results of the improved prototype design and Section 11 concludes the paper.

## 2. GMR Overview

The GMR effect was first discovered in 1988. Many different applications have been developed subsequently, including current sensors, position sensors, velocity sensors, Magnetoresistive Random Access Memory (MRAM) [9] and hard disks. The rapid development in the GMR field was recognized by the 2007 Nobel Prize in Physics.

**Figure 1.**
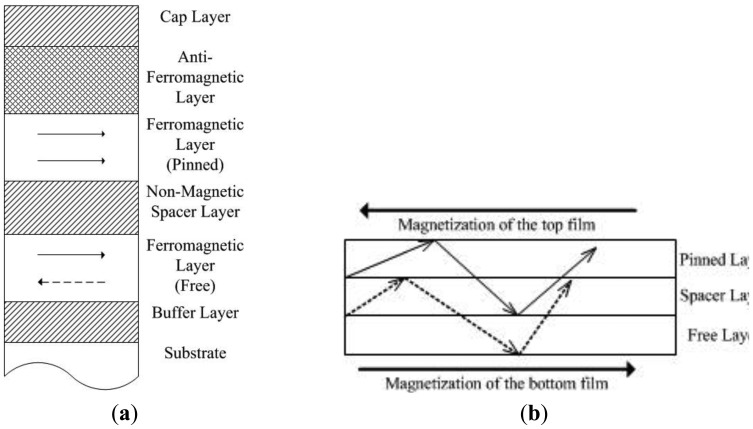
**(a)** Schematic cross-section of a GMR layered structure; (**b**) limited electron path due to scattering.

The GMR effect describes that the resistance changes according to the angle between the directions of the magnetization of adjacent layers. Although there are two kinds of inhomogeneous material systems which the GMR effect can occur in, including granular systems [15] and discontinuous multilayers [16], applications are mainly focused on the multilayers [17]. In this paper, the proposed sensor design is also implemented on a GMR with multilayer structure. Figure 1(a) shows an example a multilayer GMR structure. There is a non-magnetic spacer layer that separates two ferromagnetic layers. One of the ferromagnetic layers is pinned by an anti-ferromagnetic layer as the two layers are in contact with each other. The non-magnetic spacer layer is sufficiently thick so that the magnetic coupling does not happen on another ferromagnetic layer which is then called unpinned. Except special cases, the direction of the magnetization of the pinned ferromagnetic layer is fixed. When an external magnetic field is applied to the unpinned or free ferromagnetic layer, the angle of the magnetization direction between the two layers can be changed from a parallel to an anti-parallel alignment.

Figure 1(b) shows that the electron path is limited as the electrons scatter off the interface of the two ferromagnetic layers. It is because the magnetization direction changes the spin characteristics of the conducting electrons in magnetic materials. The current flows from left to right in the diagram. The electrons are referred to as “spin up electrons” in the top ferromagnetic layers, and electrons arereferred to as “spin down electrons” in the bottom ferromagnetic layer which is magnetized in the direction opposite to the top layer. The electrons tend to scatter off the two interfaces of the free layer and the pinned layer as the “spin up electrons”, whose path is drawn in the solid line, are entering the spin down layer, and vice versa, whose path is drawn dotted line. This effectively reduces the free path of electrons. Scattering is strong when the alignment of the two layers is antiparallel and vice versa [18]. The difference in electron spin between the pinned layer and the free layer determines the average free path. As a result, the change of resistance (ΔR/R) can be used to indicate the direction and the strength of the magnetic field (H) under measurement. The relationship between the resistance of GMR and magnetic field is well summarized in [19,20].

Compared to the Hall effect sensor and Anisotropic Magneto-resistive (AMR) sensor, the GMR sensor has the advantages of higher sensitivity, wider frequency range, smaller size, lower power consumption and relatively low cost. Despite the advantages, its applications are mainly focused on low-field environments. Therefore, a lot of research has been done to extend the linearity range of GMR sensors, such as through modeling [10] and circuit compensation [6].

## 3. Helmholtz Coil

**Figure 2.**
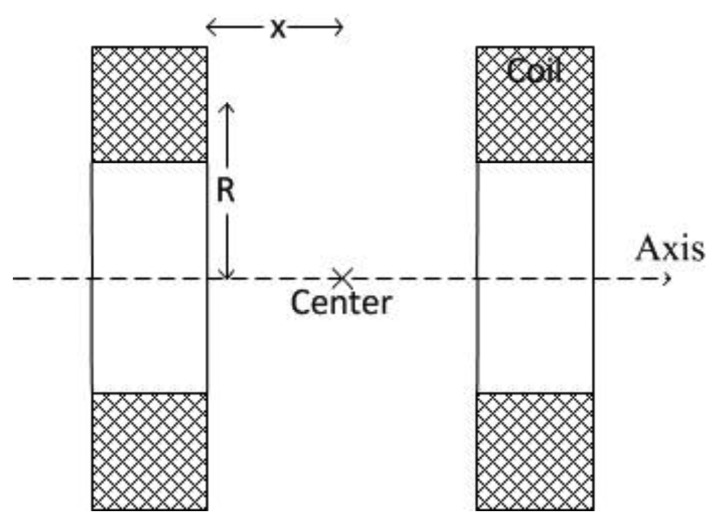
Geometric arrangement of the Helmholtz coil.

The Helmholtz coil was first invented by the physicist Hermann von Helmholtz [21]. The Helmholtz coil is defined as two identical circular coils that have same diameter and equal number of turns, placed parallel to each other along a common axis through the center of the coils distanced by the radius of the coils [22]. With the same amount of current flowing through the two coils, the magnetic field near the center of the axis would be nearly uniform. It can be guaranteed by configuring the two coils in series. The geometric arrangement of the two coils is as shown in Figure 2.

According to the Biot-Savart law, the magnetic field H_s_ generated by a single wire coil at the center point of the axis is:

(1) $${\text{H}}_{\text{S}}=\frac{\text{I}{\text{R}}^{2}}{2{\left({\text{R}}^{2}+{\text{x}}^{2}\right)}^{3/2}};$$

where R is the radius of the coil, x is the distance from the coil to the center point; and I is the current flowing through the coils.

When the two coils each contain N number of turns, the magnetic field strength is multiplied by N. As the two coils are placed equally R/2 from the center point of the axis, the magnetic field strength H_x_ generated by the Helmholtz coil at the center of the axis is:

2) (2) $${\text{H}}_{\text{X}}=\frac{{\text{NIR}}^{2}}{{\left[R^{2}+{\left(\frac{R}{2}\right)}^{2}\right]}^{3/2}};$$

which is simplified into:

3) (3) $$H_{x}=\frac{NI}{1.25^{3/2}R}.$$

The uniform magnetic field produced at the center point of the axis of the Helmholtz coil is a very useful feature, which has been utilized for immunity testing [23], calibration [24], hyperpolarization [25], and bioelectromagnetic studies [26].

## 4. Design Development

**Figure 3.**
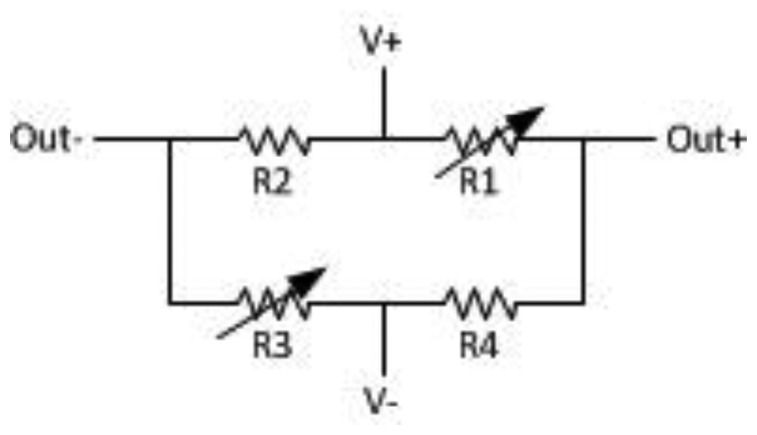
Wheatstone bridge configuration.

The GMR sensor adopted in the prototype is the NVE's AAL002-02 [27] unit whose GMR elements are configured in a Wheatstone bridge as shown in Figure 3. The sensor has an axis of sensitivity in the plane.

The two shielded resistors in the bridge are for temperature compensation. The other two resistors are affected by external magnetic field. With increasing magnetic field, the resistance decreases. The bridge output represented by Equation (4) is always unipolar:

(4) $${\text{Out}}_{+}-{\text{Out}}_{-}=\left({\text{V}}_{+}-{\text{V}}_{-}\right)\left(\frac{{\text{R}}_{3}}{{\text{R}}_{2}+{\text{R}}_{3}}-\frac{{\text{R}}_{4}}{{\text{R}}_{1}+{\text{R}}_{4}}\right)\cdot$$

**Figure 4.**
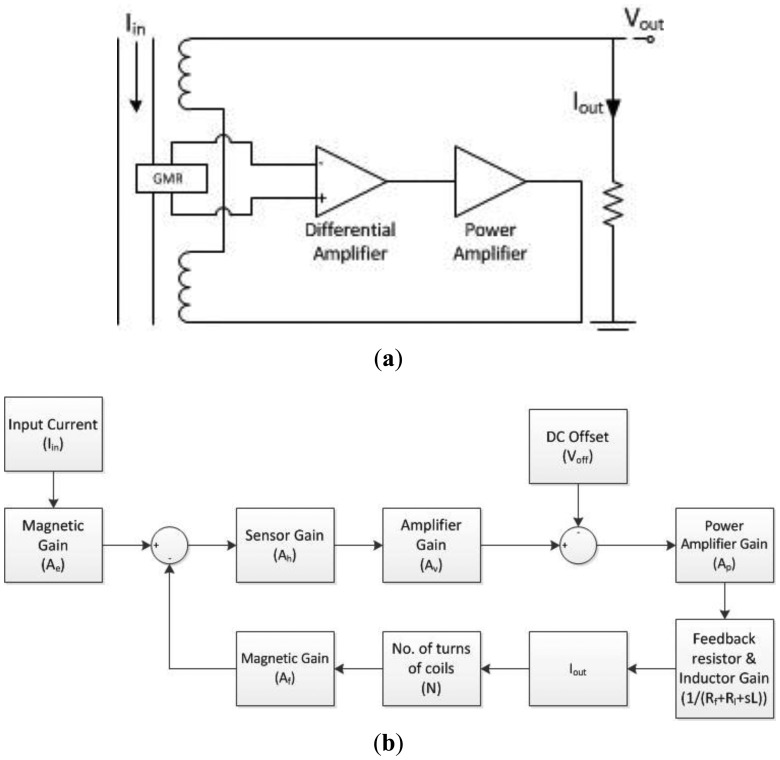
**(a)** Simplified circuit diagram; **(b)** block diagram of the system.

The simplified circuit diagram is shown in Figure 4(a). In this circuit, the GMR sensor is placed between two coils. When the input current I_in_ flows through the wire that is fixed below the sensor, the resistance of the sensor is changed by the magnetic field from the current. The change of the resistance is proportional to the field strength, and the field strength can be measured by the voltage across the differential output of the GMR sensor (Out+, Out−). The output current I_out_ can be measured accordingly. It is important to note that the two coils configured as a Helmholtz coil [19] are placed on the two sides of the GMR sensor. The orientation of the devices is so arranged to make sure the magnetic field generated by the Helmholtz coil counteracts the magnetic field produced by the current carrying wire. Therefore, the net magnetic flux through the GMR sensor is adjusted to be within the designated linear range.

The block diagram of the proposed system is shown in Figure 4(b). Assuming that the input current and the output current are denoted as I_in_ and I_out_, respectively, the transfer function of the system can be written as:

(5) $${\text{I}}_{\text{out}}=\frac{{\text{A}}_{\text{e}}{\text{A}}_{\text{h}}{\text{A}}_{\text{p}}{\text{A}}_{\text{v}}{\text{I}}_{\text{in}}-{\text{A}}_{\text{p}}{\text{V}}_{\text{off}}}{{\text{A}}_{\text{f}}{\text{A}}_{\text{h}}{\text{A}}_{\text{p}}{\text{A}}_{\text{v}}\text{N}+{\text{R}}_{\text{f}}+{\text{R}}_{\text{l}}+\text{sL}}.$$

According to Ampere's law, the magnetic gain A_e_ of the generated magnetic strength H_e_ due to the input current is:

(6) $${\text{A}}_{\text{e}}=\frac{{\text{H}}_{\text{e}}}{\text{I}}=\frac{1}{2\pi \text{r}}$$

where r is the distance from the center of the wire to the sensor.

Since the resistance of the GMR sensor changes with the net magnetic field strength H_h_, the resistance can be obtained by the voltage difference across the differential output of the sensor. The gain of the output voltage V_h_ to the generated magnetic strength is:

(4) $${\text{A}}_{\text{h}}=\frac{{\text{V}}_{\text{h}}}{{\text{H}}_{\text{h}}}.$$

In words, the output voltage is amplified by a differential amplifier with a fixed gain of A_v_. As the output of the GMR sensor is unipolar, it is necessary to bias the GMR sensor to produce bipolar output. Current biasing is used in the design as the current can simply be controlled by a voltage offset V_off_. The V_off_ controls the output voltage of the differential amplifier so as to maintain a constant current in the feedback coil. It effectively creates a magnetic pseudo-zero point for the GMR sensor.

A power amplifier with a gain of A_p_ is then used to provide sufficient current to generate the counteracting magnetic field. The output current I_out_ is limited by two components, a series resistor and an inductor (the Helmholtz coil – air coil). A series resistor R_f_ is used to measure the actual output current I_out_ and to prevent the current from overwhelming the circuit. The feedback Helmholtz coil is modeled as an inductance L with resistance of R_l_. The gain A_f_ of the output current I_out_ to the voltage output V_p_ of the power amplifier is given by:

(5) $$\frac{{\text{I}}_{\text{out}}}{{\text{V}}_{\text{p}}}=\frac{1}{{\text{R}}_{\text{f}}+{\text{R}}_{\text{l}}+\text{sL}}$$

where s is jω in the s plane.

The two identical air coils are configured as a Helmholtz coil and can create a region of nearly uniform magnetic field when they are separated by their radius R. Therefore, when the GMR sensor is placed in between the two coils, the error in measurement caused by inaccurate positioning of the GMR sensor is reduced. The axis of the GMR sensor is parallel to the axis of the Helmholtz coil so as to maximize the cancellation effect.

When a current flows through the two coils, the magnetic field strength is amplified by the number of turns of the coils (N). The gain A_f_ of the feedback magnetic strength H_f_ to the output current is:

(6) $${\text{A}}_{\text{f}}=\frac{{\text{H}}_{\text{f}}}{{\text{NI}}_{\text{out}}}={\left(\frac{4}{5}\right)}^{3/2}\frac{1}{\text{R}}.$$

The feedback coils generate a magnetic field counteracting the magnetic field produced by the input current. The net magnetic flux is reduced and in this sense the linear range of the GMR sensor is extended. With a sufficiently high gain, the output current is then proportional to the input current. For ease of measurement, the output current is measured by the voltage V_out_ across a series resistor. Then the transfer function can be rewritten as

(7) $${\text{V}}_{\text{out}}=\frac{{\text{A}}_{\text{e}}{\text{A}}_{\text{h}}{\text{A}}_{\text{p}}{\text{A}}_{\text{v}}{\text{I}}_{\text{in}}-{\text{A}}_{\text{p}}{\text{V}}_{\text{off}}}{{\text{A}}_{\text{f}}{\text{A}}_{\text{h}}{\text{A}}_{\text{p}}{\text{A}}_{\text{v}}\text{N}+{\text{R}}_{\text{f}}+{\text{R}}_{\text{l}}+\text{sL}}{\text{R}}_{\text{f}}\cdot$$

## 5. Design Characteristics

Preliminary experiments have been conducted to study the behaviors of the prototype sensors based on the design mentioned in the previous section. The following characteristics of the proposed GMR sensors are observed:

1. Larger A_e_ can increase the sensitivity. This implies that the current-carrying wire should be fixed as near to the GMR sensor as possible.
2. When the product of A_f_, A_v_, and N is much larger than the inductive impedance (sL), the inductance has minimal effect on the system and this can improve the frequency response of the design.
3. A_h_ is the characteristic of the GMR sensor that is not controllable. A high A_h_ is more suitable for sensing as the sensitivity of the sensor is higher.
4. As long as the product of V_off_ and A_p_ remains sufficient to bias the GMR sensor, the exact value of V_off_ and A_p_ is not important. However, a larger value of A_p_ would lead to a smaller cutoff frequency. Therefore, a small A_p_ is preferred.
5. The combined resistance of R_f_ and R_l_ should be chosen carefully to protect the power amplifier from damaging by over-current.

**Figure 5.**
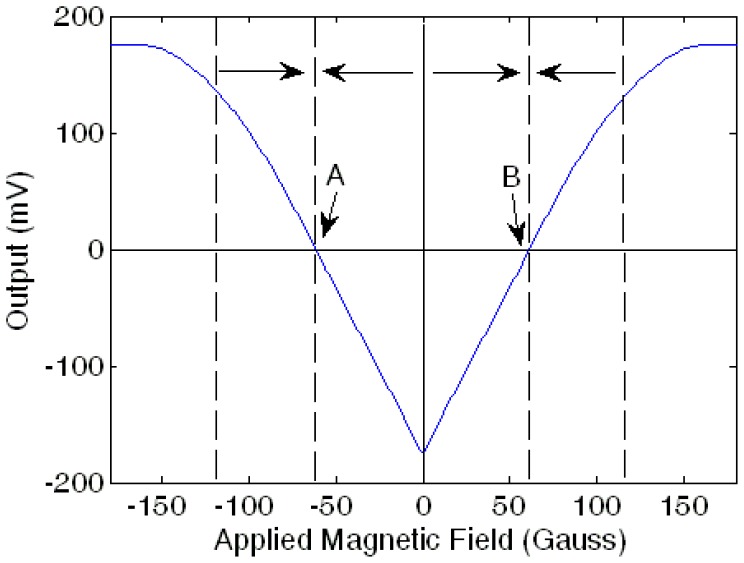
Offset output of the sensor.

Changing the direction of the coils does not affect the functionality of the sensor. This is because the magnetic field generated by the Helmholtz coil is counteracting the magnetic field of the input current when the gain of the sensor is high enough. Depending on the direction of the coils, the negative voltage offset drives the sensor until it is stable at the pseudo-zero point close to either point A or point B shown in Figure 5. It implies that the counteracting magnetic field is opposite to the output of the sensor which is proportional to the field of the input current. The orientation of the coils would only affect the sensor output polarity, but this can easily be calibrated during sensor testing.

## 6. Design Implementation

A prototype current sensing circuit is implemented. The NVE AAL002-02 GMR sensor unit [11] is used as the magnetic field sensing element, which has good performance on nonlinearity and hysteresis. The differential amplifier and power amplifier adopted are the Texas Instrument INA118 and OPA564, respectively. The main parameters of the amplifiers and the GMR sensor are listed in Table 1. It shows that the amplifier has a usable frequency response up to 500 kHz, and the GMR sensor consumes very little power in its resistance.

**Figure 6.**
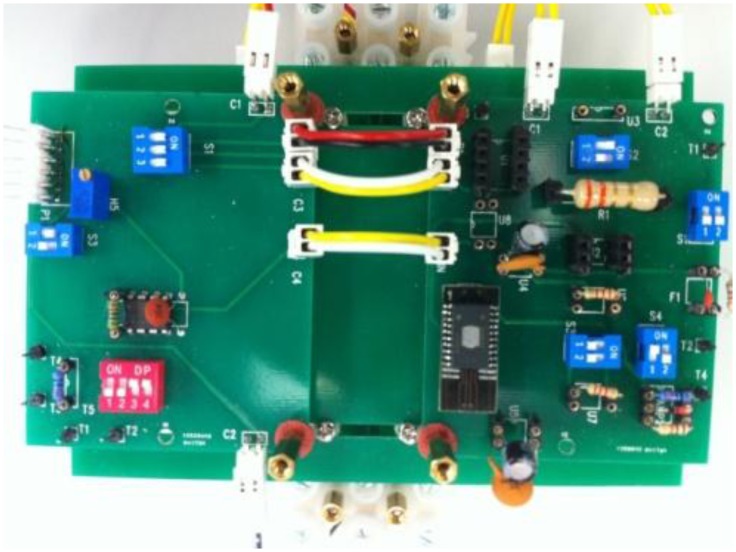
Photo of the prototype current sensor.

The geometric parameters and the electrical parameters are shown in Tables 2 and 3 respectively. A photograph of the prototype unit is shown in Figure 6.

## 7. Performance Results

A series of tests has been conducted to evaluate the performance of the prototype GMR sensor in linearity range, frequency response and power consumption. They are discussed as follows.

### 7.1. Linearity

**Figure 7.**
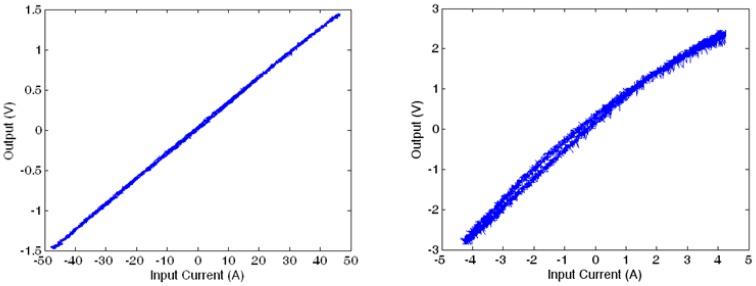
Linearity of the current sensor (**a**) with Helmholtz coil and; (**b**) without Helmholtz coil.

The linearity range of the proposed GMR current sensor is important since the design is expected to produce accurate measurement results over the useful working range. In order to evaluate the linearity range of the prototype sensor, a high power resistive load is used for the testing. The current through the sensing circuit varies from −45 A to +45 A and the corresponding output voltages are measured. The result is shown in Figure 7(a) and the wide range linearity is well demonstrated. For comparison purposes, Figure 7(b) shows the results of the same design, but with the feedback Helmholtz coil disconnected. It is clearly shown that the range of measurement is only one tenth of the design with Helmholtz coil and yet its output is not linear. The correlation coefficient is 0.9999 which implies a strong relationship within the test current range. On the contrary, if the Helmholtz coil is removed, the current sensor would saturate at a much lower current range of around 9 A (for comparison purpose, the measured current values shown in Figure 7(b) are offset by the mean current value).

### 7.2. Frequency Response

**Figure 8.**
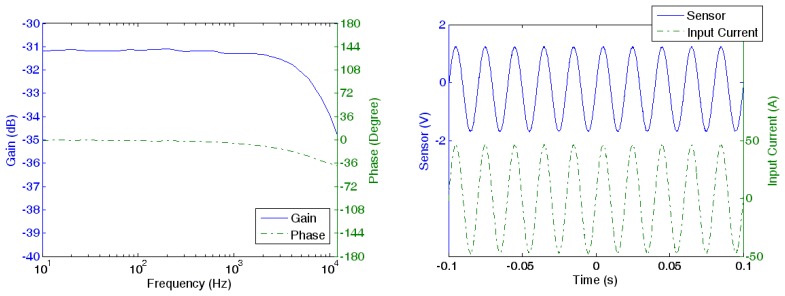
(**a**) Frequency response of the sensor; (**b**) waveform of the input current and the output of sensor at 50 Hz.

A gain-phase analyzer, Agilent's model 4194A, is used to study the frequency response of the prototype current sensor. By comparing the input current and output current, the gain and the phase between the two signals are obtained. The cutoff frequency of the sensor is at 10 kHz as shown in Figure 8(a). The early cutoff is due to the increase of the impedance of the feedback Helmholtz coil at higher frequencies. Thus the feedback current decreases with the frequency. Although the cutoff frequency is much lower than the cutoff frequency of the amplifiers and the GMR sensor, a cutoff frequency of 10 kHz is acceptable for PQ monitoring in building LV electrical network as the power harmonics measurement is up to the 63rd harmonics (3,150 Hz). The waveforms of the input current and the output current of the sensor at 50 Hz are shown in Figure 8(b).

### 7.3. Power Consumption

**Figure 9.**
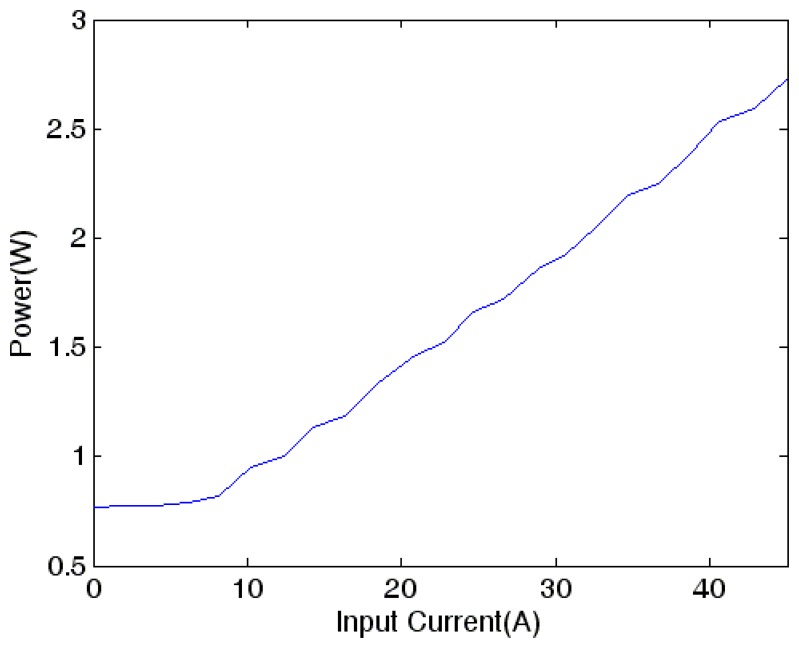
Power consumption *vs.* the input current.

The power consumption of the prototype current sensor *vs.* the input current is shown in Figure 9. It is noted that the current sensor consumes over 2.7 W when measuring 45 A current. That means a comparatively high power is needed to counteract the magnetic field produced by the current-carrying wire so that the output linearity can be maintained at high current values. As the sensor needs voltage offset current to bias the GMR sensor, the current sensor would consume 0.7 W even if the input current is zero. Moreover it is found that a heat sink is necessary to dissipate the heat produced by the power amplifier, otherwise drift in operating characteristics caused by temperature rise is appreciable.

## 8. Automatic Gain Control for the GMR Current Sensor

In the current design, although the linear range of the prototype current sensor is extended significantly, neither the frequency response nor the power consumption are optimized. One obvious reason is that a sufficiently large current is needed to sustain the gain to counteract the magnetic field under measurement so that the linear range can be extended.

To optimize the frequency response and the power consumption, an intelligent control is implemented to adjust the gain of the prototype sensor so that the linear range of the current sensor is fully utilized. One advantage is that the sensor does not need to provide the same high power if the input current is small. In this way, the product of gain and bandwidth is fixed which results in a wider frequency bandwidth and a lower power consumption.

**Figure 10.**
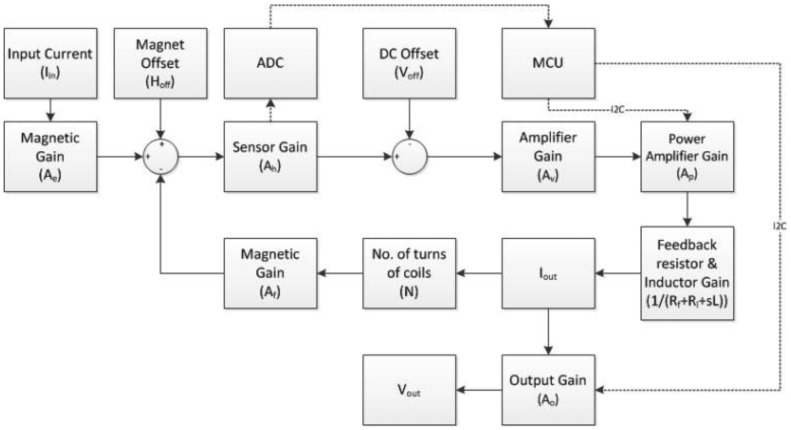
Block diagram of the improved design.

In addition to optimizing the frequency response and the power consumption, a frequency-compensated inductor is added to the circuit to compensate for the decrease in current caused by the Helmholtz coil at higher frequencies. The block diagram of the improved design is shown in Figure 10.

The transfer function of the improved design can be written as:

(8) $$\left[\left({\text{A}}_{\text{e}}{\text{I}}_{\text{in}}-{\text{A}}_{\text{f}}\text{N}{\text{I}}_{\text{out}}+{\text{H}}_{\text{off}}\right){\text{A}}_{\text{h}}-{\text{V}}_{\text{off}}\right]\frac{{\text{A}}_{\text{v}}{\text{A}}_{\text{p}}}{{\text{R}}_{\text{f}}+{\text{R}}_{\text{l}}+\text{sL}}={\text{I}}_{\text{out}}.$$

As the operating point of the original design is determined solely by the current driven by the DC offset, the operating point would be changed if the gain is changed. Therefore, a permanent magnet is used to offset the GMR sensor and the DC offset is only used to prevent a net current from feeding back to the GMR sensor. The magnetic field H_off_ of the permanent magnet and DC offset V_off_ are adjusted as:

(9) $$A_{h}H_{\text{off}}-V_{\text{off}}=0.$$

The permanent magnet offsets the GMR sensor to the midpoint of linear range so that the full linear range can be utilized. Then the transfer function can be simplified to:

(10) $$\frac{{\text{A}}_{\text{e}}{\text{A}}_{\text{h}}{\text{A}}_{\text{p}}{\text{A}}_{\text{v}}{\text{I}}_{\text{in}}}{{\text{A}}_{\text{f}}{\text{A}}_{\text{h}}{\text{A}}_{\text{p}}{\text{A}}_{\text{v}}\text{N}+{\text{R}}_{\text{f}}+{\text{R}}_{\text{l}}+\text{sL}}={\text{I}}_{\text{out}}.$$

**Figure 11.**
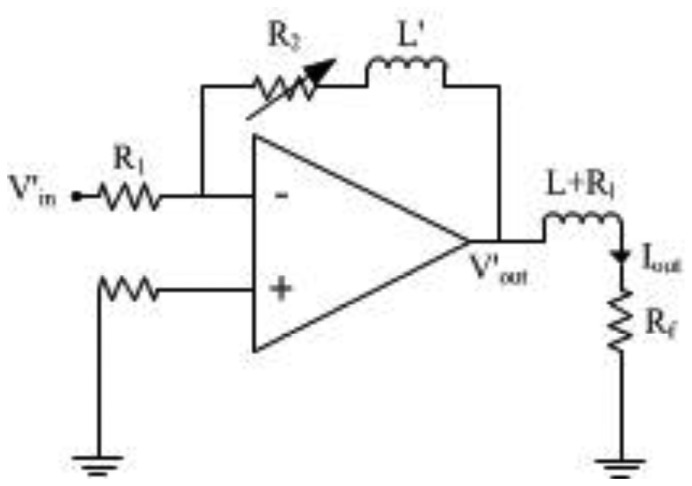
The circuit of the power amplifier.

Another improvement to the design is that the dynamic control of the gain A_p_ of the power amplifier is controlled by a MCU. The MCU monitors the output of the GMR sensor via an Analogue-to-Digital Convertor (ADC). When the output is out of range continuously, the MCU changes the gain by adjusting a digital resistor via I2C. The circuit for the power amplifier in the improved design is shown in Figure 11.

In this design the gain of the power amplifier can be adjusted according to the linear range required. When the output is equal to or below 90 % of a particular range, a lower linear range can be chosen, and *vice versa*. This is accomplished through adjusting R_2_ in Figure 11. Besides the control of the gain, there is an inductor (indicated as L′ in Figure 11) added to the circuit to compensate the decrease in current as the frequency increases. Therefore, the gain A_p_ of the power amplifier is written as:

(11) $${\text{A}}_{\text{p}}=-\frac{{\text{R}}_{2}+\text{s}{\text{L}}^{\prime }}{{\text{R}}_{1}}.$$

The feedback current is then changed to:

(12) $${\text{I}}_{\text{out}}=-{{\text{V}}^{\prime }}_{\text{in}}\frac{{\text{R}}_{2}+\text{s}{\text{L}}^{\prime }}{{\text{R}}_{1}\left({\text{R}}_{\text{f}}+{\text{R}}_{\text{l}}+\text{sL}\right)}.$$

By choosing L′, and R_1_ and R_2_ correctly, the inductor L′ can compensate the current I_out_ as the frequency of the input current increases. R_1_, R_2_ and L′ should fulfill the following two conditions.

(13) $${\text{R}}_{2}=\text{C}{\text{R}}_{1}\left({\text{R}}_{\text{f}}+{\text{R}}_{\text{l}}\right);$$

and:

(14) $${\text{L}}^{\prime }=\text{C}{\text{R}}_{1}\text{L};$$

where C is a non-zero arbitrary constant.

The transfer function is simplified to:

(15) $$\frac{{\text{A}}_{\text{e}}{\text{A}}_{\text{h}}{\text{A}}_{\text{v}}{\text{CI}}_{\text{in}}}{1+{\text{A}}_{\text{f}}{\text{A}}_{\text{h}}{\text{A}}_{\text{v}}\text{N}}={\text{I}}_{\text{out}}$$

It can be seen that the output current is independent of the frequency of the input current. And by controlling the constant C via the digital resistor R_2_, the output current can be adjusted conveniently. As the output current is dynamically changed by the MCU, the output signal should be adjusted accordingly so that the output signal can represent the true value of the input current. Therefore, the output voltage V_out_ is adjusted by an amplifier with digitally controlled gain A_o_ as:

(16) $$\frac{{\text{A}}_{\text{e}}{\text{A}}_{\text{h}}{\text{A}}_{\text{o}}{\text{A}}_{\text{v}}\text{C}{\text{I}}_{\text{in}}}{1+{\text{A}}_{\text{f}}{\text{A}}_{\text{h}}{\text{A}}_{\text{v}}\text{N}}={\text{V}}_{\text{out}}$$

## 9. Improved Design Implementation

The improved design of the current sensor is implemented with an independent MCU incorporated. In practical applications, the MCU of a PQ meter / smart meter can be configured to control the current sensor accordingly. The MCU adopted is the Texas Instrument model no. TMS320F28335. The MCU has a built-in 12-bit ADC. The two digital resistors that operate on 10-bit from 0 Ω to 20 kΩare Analog model no. AD5293. The MCU communicates with the resistors using I2C protocol. As the two resistors are connected in a daisy chain, the change of the resistance of the two resistors can be triggered simultaneously. Therefore, the time taken for the two resistors to stabilize after a change of gain is minimal. Additional electrical parameters of the improved design are tabulated in Table 4.

**Figure 12.**
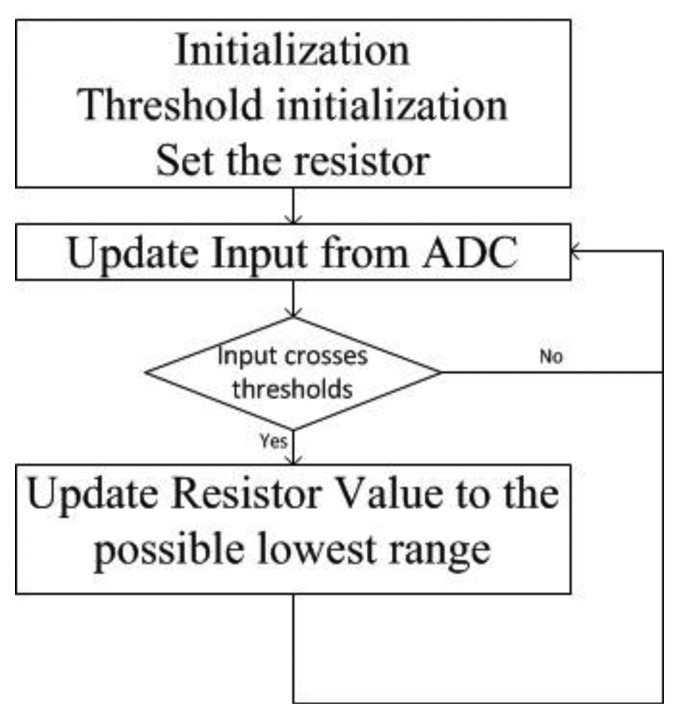
Control Logic.

Figure 12 shows the control logic of the MCU. During startup, the MCU initializes the hardware operation, including the digital resistors, to measure at the maximum linear range. As there are three effective range selections, there are two thresholds. After initialization, the MCU keeps updating the current value of the input from the ADC. Whenever the input current crosses either one of the two thresholds, the resistor is set for the lowest possible gain of the counteracting field. Then the MCU will check the ADC input again and the procedures depicted in Figure 12 are repeated.

## 10. Performance Results

### 10.1. Linearity

**Figure 13.**
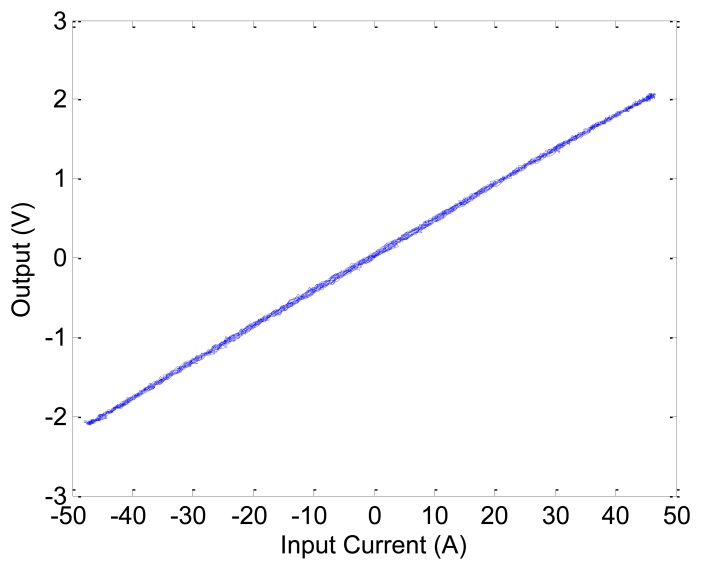
Linearity of the improved design.

It can be seen that the improved design still has a linear range extended to ±4 5 A, as shown in Figure 13.

### 10.2. Frequency Response

**Figure 14.**
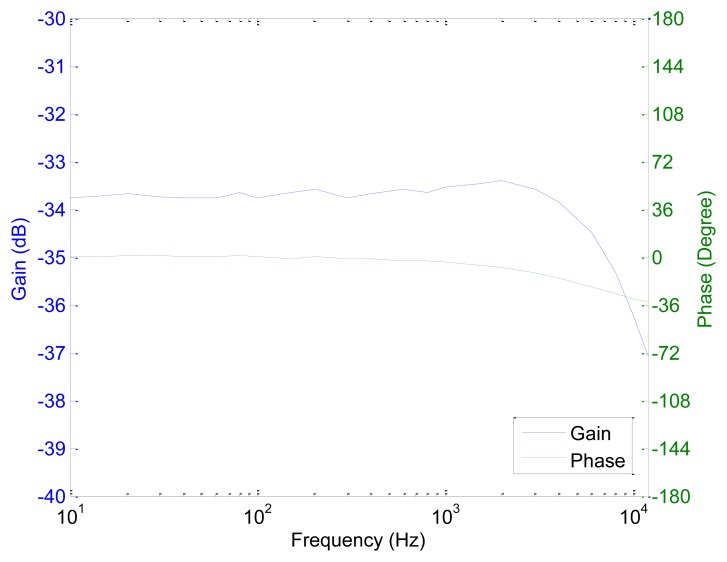
Frequency response of the improved design.

The improved prototype GMR current sensor can measure up to a cutoff frequency of 12 kHz (20% increase as compared to the original design) as shown in Figure 14. Although the improvement is not significant enough to expand the application of the current sensor, it shows that the design can be optimized for a wider frequency range.

### 10.3. Power Consumption

**Figure 15.**
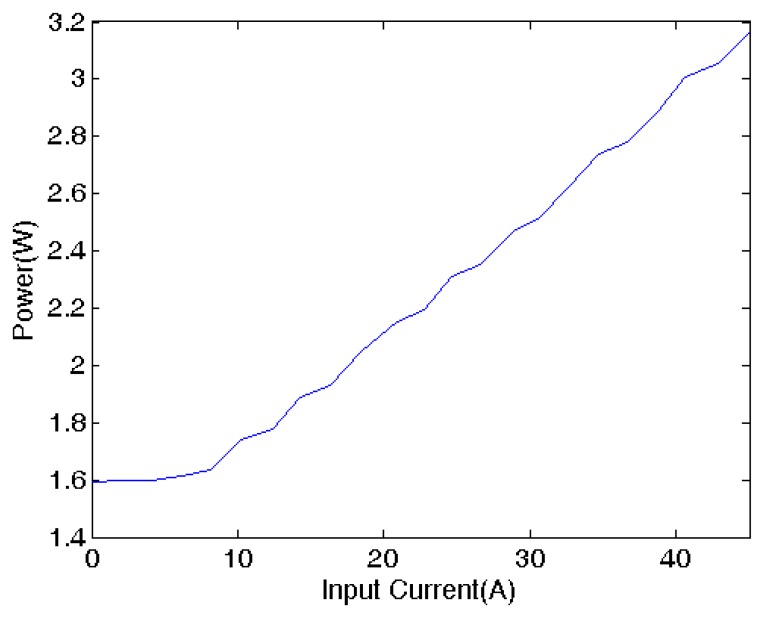
Power consumption *vs.* the input current of the improved design.

Figure 15 shows the power consumption of the improved prototype GMR current sensor *vs.* the input current. The current sensor consumes around 3.2 W when measuring a current of 45 A. Compared to the previous design, an extra 0.5 W is used for the additional circuit components,including the conditioning circuit for ADC, the output signal amplifier and the digital resistors. It is envisaged that with components carefully chosen, further power consumption reduction can be achieved.

## 11. Conclusions

This paper presents a circuit design technique to extend the current measurement range of a GMR sensor from 9 A (unipolar) to ±45 A. By using a Helmholtz coil, a magnetic field is generated to counteract the magnetic field produced by the current carrying wire. In addition, an automatic dynamic scaling function is implemented by using a microcontroller unit to control the counteracting gain so that the frequency response and the power consumption can be optimized. This is very useful in high power electronic circuit designs requiring current measurement, such as power inverters and distribution circuit current measurement in LV electrical network for future building microgrid applications.

## Figures and Tables

**Table 1.** Main parameters of the sensor and the amplifiers.

**Parameter**	**Value**	**Unit**

**AAL002-02**		
Saturation field	1194	A/m
Linear Range	[119 836]	A/m
Sensitivity	[37.7 53.8]	μV/V/Am^−1^
Resistance	5.5	kΩ
Operating Frequency	DC to 1	1MHz
Nonlinearity (unipolar)	2	%
Hysteresis (uniploar)	2	%

**INA118**		

Cutoff Frequency, A_v_ = 10	500	kHz

**OPA564**		

Max. DC current	1.5	A
Gain Bandwidth Product	17	MHz

**Table 2.** Geometric parameters.

**Parameter**	**Value**	**Unit**
Distance r	0.003	m
Number of turn N	300	
Radius of Coil R	0.0065	m
Distance between Coils	0.011	m

**Table 3.** Electrical parameters.

**Parameter**	**Value**	**Unit**
Gain A_h_	[0.679 0.950]	Vm/A
Gain A_p_	1	
Gain A_v_	10	
Inductance L	515	µH
Resistance of Inductor L	3.69	Ω
Series Resistor R_f_	3.3	Ω
Voltage Offset	2.95	V
Power supply	±9	V

**Table 4.** Additional electric parameters.

**Parameter**	**Value**	**Unit**
Resistor R_1_	0.003	Ω
Resistor R_2_	300	Ω
Inductor L′	0.5	H
